# Towards a new model for producing evidence-based guidelines: a qualitative study of current approaches and opportunities for innovation among Australian guideline developers

**DOI:** 10.12688/f1000research.19661.1

**Published:** 2019-06-24

**Authors:** Steve McDonald, Julian H. Elliott, Sally Green, Tari Turner

**Affiliations:** 1 Cochrane Australia, School of Public Health and Preventive Medicine, Monash University, Melbourne, VIC, 3004, Australia

**Keywords:** Clinical guidelines, Updating guidelines, Living guidelines, Qualitative research, Interviews

## Abstract

**Background:** Many organisations in Australia undertake systematic reviews to inform development of evidence-based guidelines or would like to do so. However, the substantial resources required to produce systematic reviews limit the feasibility of evidence-based approaches to guideline development. We are working with Australian guideline developers to design, build and test systems that make creating evidence-based guidelines easier and more efficient.

**Methods:** To understand the evidence needs of guideline developers and to inform the development of potential tools and services, we conducted 16 semi-structured interviews with Australian guideline developers. Developers were involved in different types of guidelines, represented both new and established guideline groups, and had access to widely different levels of resources.

**Results:** All guideline developers recognised the importance of having access to timely evidence to support their processes, but were frequently overwhelmed by the scale of this task. Groups developing new guidelines often underestimated the time, expertise and work involved in completing searching and screening. Many were grappling with the challenge of updating and were keen to explore alternatives to the blanket updating of the full guideline. Horizon-scanning and evidence signalling were seen as providing more pragmatic approaches to updating, although some were wary of challenges posed by receiving evidence on a too-frequent basis. Respondents were aware that new technologies, such as machine learning, offered potentially large time and resource savings.

**Conclusions:** As well as the constant challenge of managing financial constraints, Australian guideline developers seeking to develop clinical guidelines face several critical challenges. These include acquiring appropriate methodological expertise, investing in information technology, coping with the proliferation of research output, feasible publication and dissemination options, and keeping guidance up to date.

## Introduction

Many organisations in Australia and internationally undertake and use systematic reviews to inform development of clinical practice guidelines. This process is critical for translating the results of research into evidence-informed decision making and clinical practice to ensure the best possible outcomes for patients ^1^. It is essential that this process is both efficient and cost-effective, particularly given the significant time, effort and resources spent developing guidelines. In Australia, for example, by 2015 around 600 locally developed guidelines were included in the National Health & Medical Research Council (NHMRC) guidelines portal ^2^.

In 2016, in response to concerns around the trustworthiness of Australian guidelines to inform decision making, NHMRC released a consultation paper ‘Better informed health care through better clinical guidelines’ ^3^. The paper identified several challenges facing clinical guidelines in Australia, including concerns over quality, lack of investment in information technology, and obsolescence. These concerns are compounded by the resources required for research synthesis and the sheer volume of research now available.

Systematic reviews are the gold-standard approach for synthesising research evidence and should thus be the foundation of guidelines, but the substantial time and resources required to produce them potentially limit their contribution and uptake into clinical guidelines ^4^. Although this barrier has been partially ameliorated in recent years by the explosion in the number of systematic reviews being published ^5^ (allowing the focus to shift away from undertaking systematic reviews from scratch) guideline developers now have to contend with the problem of keeping up to date with, and making sense of, the burgeoning global research output.

Cochrane is a leading international organisation dedicated to ensuring that reliable evidence informs all levels of decision making in health. The routine use of systematic reviews as the evidence base for guidelines has led to numerous collaborations between Cochrane and various guideline groups to facilitate the efficient and more rapid inclusion of Cochrane evidence ^6, 7^.

In a project funded by Cochrane and NHMRC, we are working to develop new tools and systems that will substantially reduce the time and resources required to undertake rigorous systematic reviews ^8^. Our team is working on text mining technologies, machine learning systems and crowd-sourcing to improve the efficiency of study identification, citation screening and data extraction. We are also investigating other tools like automated evidence delivery services that can provide easy access to new, relevant evidence.

In this study we sought to inform this work by interviewing Australian guideline developers with the aim of understanding their evidence needs and the kinds of tools and services that will enable them to most efficiently access, produce and use reviews of research evidence in guideline development.

## Methods

We aimed to understand the evidence needs of guideline developers by exploring how guideline groups manage processes for identifying, appraising and synthesising relevant evidence, and what the implications for these processes are of potential new technologies to support guideline development. We used a qualitative approach (semi-structured interviews) to explore the experiences of guideline developers and to gauge their receptivity to new ways of managing their evidence needs.

Ethics approval was provided by Monash University (CF16/1950 – 2016000999). Reporting of the methods and results comply with the consolidated criteria for reporting qualitative studies (COREQ) checklist ^9^. A completed COREQ checklist is available ^10^.

### Participants

In 2016, following the release of the consultation paper on better guidelines ^3^, NHMRC held a series of forums for guideline developers to share knowledge and expertise in guideline development. A purposive sample of guideline developers who attended these forums was invited to participate in an interview. The sample captured participants and organisations with diverse levels of expertise and approaches to guideline development. Many of them were familiar with the interviewers and their areas of research as a result of attending earlier NHMRC forums and other similar meetings.

Participants were emailed invitations to participate in an interview, together with a brief project synopsis and an Explanatory Statement. Participation was voluntary. Acceptance of the invitation to participate in the interviews was taken as evidence of informed consent. No one approached to participate declined to be interviewed. Participants gave their consent to be interviewed on the understanding that data would be reported in de-identified summary form in which no individuals could be identified.

### Data collection

Data were collected using a semi-structured interview with guided questions, conducted by telephone or in person. Interview guides are available as *Extended data* ^10^. The interview schedule and questions were developed by S.M. and T.T. The interviews began with participants describing their experience and involvement with guidelines. The questions then explored several issues: contrasting approaches to guideline development adopted by different guideline groups; specific challenges in developing, publishing and updating guidelines; processes or steps where technical capacity is seen as a barrier; and potential advantages and disadvantages of technology in facilitating more efficient guideline development. Interviewees were encouraged to focus on how their current approach to developing guidelines could be more efficient, and what they saw as the main opportunities and challenges to achieving this.

The question(s) introducing the main issues were the same for each interview, with follow-up questions tailored according to individual responses. Six interviews (with Melbourne-based guideline developers) took place in person and 10 were conducted by telephone. Most interviews lasted between 30–40 minutes. The first three face-to-face interviews were conducted jointly by S.M. (MA; male) and T.T. (PhD; female), with S.M. conducting the remaining interviews. Both S.M. and T.T. are senior research fellows familiar with guideline development processes, and are experienced systematic reviewers and with extensive experience in qualitative research.

In addition to taking detailed notes during the interview, interviews were audio-recorded following the consent of the interviewees. Most interviews were one-on-one, but four interviews were with two participants from the same guideline organisation.

### Data analysis

Notes taken during the interviews were written up and the audio files transcribed using the transcription service Temi. We used NVivo for Mac 11 to analyse qualitative data and extract quotes. Thematic analysis of the detailed interview notes was undertaken using open coding to identify key concepts that were organised into emerging themes. S.M. undertook the primary data analysis. The initial coding of themes was reviewed by T.T. and together the final conceptual development of themes was agreed. The recordings and transcriptions of the interviews were regularly consulted for clarification and for extraction of quotes. Interviewees were not provided with transcriptions for comment or correction, and no repeat interviews were conducted.

## Results

### Respondents

We conducted 16 interviews with Australian guideline developers in August and September 2016. The sample included developers responsible for producing different types of guidelines (e.g. specific versus broad scope), representing both new and established guideline producers with access to widely different resources. The sample was sufficiently large that by the final interviews no new themes were emerging and data saturation was reached.

Organisations represented included professional colleges and societies (n=3); national bodies for specific diseases or conditions (n=6); specialist, not-for-profit guideline developers (n=1); government-funded guidelines initiatives (n=3); and academic collaborations and partnerships (n=3). Most of these organisations have a long-standing and ongoing involvement in producing guidelines; the participants we interviewed from these organisations tended to have greater technical and process expertise, and thus were able to provide extensive insights into the challenges of managing comprehensive, dedicated guideline efforts. In contrast, the groups involved in developing one-off guidelines had to contend with different concerns, typically relating to funding, time constraints and technical capacity.

All the organisations interviewed stated a commitment to producing reliable, evidence-based guidance. In practice, this was characterised by organisations that were either actively pursuing endorsement of their guidelines from NHMRC (as a marker of their evidence-based status) or were aware of this option but lacked sufficient time and resources required to pursue approval.

The two higher order themes identified focused on current challenges and new approaches to guideline production. These themes were further divided into subthemes based around specific challenges and approaches. 

### Challenges in guideline production

The diversity of organisations and guideline groups represented in the interviews was reflected in the substantial variation in approaches and models described for producing guidelines. However, irrespective of the approach taken or the resources and expertise available, guideline developers faced similar challenges, albeit on differing scales. The key challenges to emerge were capacity restraints around technical and methods expertise; resource constraints around funding and timelines; managing relations with external contractors; dealing with growing volumes of research evidence; publishing arrangements; and updating.

***Technical expertise.*** Constraints imposed by having limited access to methods expertise, whether internal or external, was a common theme. Guideline groups found it difficult to find suitably skilled staff who could work autonomously; this in turn affected the quantity of synthesis work that could be undertaken at any one time.

*“When we were trying to look for casual staff, there didn't seem to be many people around who had experience in running systematic reviews and could help us with a fairly high level of autonomy, and particularly in the diagnostic reviews. It's just a real shortage of skills.” [Participant (P)1]*

Compounding limited internal capacity was the under-appreciation by senior staff within the organisation of what is required to develop high quality guidelines.

*“Any kind of guide to helping college boards and the like understand what is involved in developing a clinical practice guideline, including resourcing and some idea of cost, would be enormously helpful. I'll just sit at the board meeting and someone will say, ‘We need a guideline on […].’ Well, you know, they have got no concept of what a high quality thing that might be.” [P2]*

The lack of methods expertise and infrastructure to support guideline activities in-house was more keenly felt by smaller guideline groups for whom it was unrealistic to have methodologists on staff, and who relied on the contributions of volunteer guideline group members.

Some guidelines were developed almost entirely on a voluntary basis by clinicians who had little or no input from specialists in evidence synthesis or medical writing, and who lacked the in-depth knowledge of how to derive recommendations using a standardised approach. In one case this led to a series of guidelines from the same organisation all being developed in different ways.

*“They were meant to [be done in the same way] but what ended up happening is that because each group was different with a different chair and different makeup … they kind of veered off and did their own thing.” [P3]*

Even in circumstances where outsourcing or buying-in expertise was possible, because the guideline work was adequately funded, respondents acknowledged that a degree of internal expertise was needed to communicate requirements and assess the work or service supplied.

*“You can definitely have a more effective dialogue with your outsourced reviewers if you have a good feel for the process yourself and if you can use the terminology.” [P4]*

***Resource constraints.*** A recurring theme was the trade-off between finite resources and rigorous methods. Even for guideline groups able to out-source some activities, the high costs associated with some of these tasks (e.g. conducting systematic reviews to underpin evidence-based guidelines) were often a deterrent. High costs were also perceived to undermine a commitment to rigorous methods, especially when there is a perception that there is little value to be gained. One respondent observed the tension between the pragmatic approach that was acceptable to the guideline group and the gold-standard approach insisted on by the technical specialists, for example by requiring two people to screen all citations and independently extract data.

*“They wanted to maintain a certain level of quality from their end, which was fine, but with our guideline reviews, we've tended to do that with one person just because of the resource implications and the need to do things quickly and efficiently.” [P1]*

The perceived value in pursuing rigorous methods versus the opportunity costs in terms of effort and resources led a couple of respondents to question whether the thorough, gold-standard approach to developing guidelines (as is required for NHMRC endorsement of guidelines, for example) delivered a ‘better’ guideline than one developed more pragmatically.

*“I'd say the evidence searching and GRADE are the big areas for us, and also that tipping point of how much effort do we put into some processes that aren't necessarily going to change what we currently have as an output. […] I mean, we could go to the nth degree of thoroughness, but I'm not sure that that's going to win us more money or keep us afloat, or meet the needs necessarily of people that use us.” [P5]*

Related to this, the time taken to complete guidelines, and the implications of this for the currency of the guidance, was another common concern, with all steps in the process (searching, screening, synthesis, public consultation) seen as time-consuming. As before, some guideline group members questioned the value of pursuing a rigorous approach.

*“We would never publish anything if we did that [follow a rigorous, evidence-based development process.]” [P6]*
*“As [our chair of the board] says, ‘We never really get it wrong’. So despite the fact that we are not having this absolute thorough, rigorous sort of review of the evidence, nobody ever writes to us and says ‘Oh my God, that’s completely wrong’.” [P6]*

***Using external suppliers or contractors.*** Large guideline groups that had the resources to outsource synthesis work acknowledged the benefits this brought – “the professional help we had … was certainly a major enabling factor” – but also described some challenges. Aside from the costs discussed above, two other issues were mentioned: problems caused when contracted providers changed part-way through, and the difficulties in accessing data and information from contractors.

The sometimes precarious nature of arrangements with external suppliers was exemplified by stories of work being started by one supplier and completed by another. As well as being a threat to the continuity of the guideline development process, it highlighted the potential lost opportunity to build internal capacity. At the very least, there were implications for workload and opportunity costs associated with wasted effort trying to rectify things. One participant commented:

*“You know what it's like picking up somebody else's research work. It's extremely difficult. I was looking in very great detail at the output and I can tell you that was one of the hardest pieces of work that I've ever done because I really had to be absolutely across everything that they’d done.” [P7]*

The second issue, of accessing the backend systems or data generated by contractors so that information can be verified or easily reused, was also problematic.

*“A lot [of the work] was done in Excel and Word, and while that’s not completely unreplicable, the problem is all of that data entry – if you want to do it again – you have to do it again.” [P7]*

The implications of limited flows of information and data between guideline developer and contractor may only become apparent later. One participant highlighted the knowledge lost as a result of outsourcing during previous editions of the guideline (e.g. no access to files of original decisions regarding bias assessments), making the process of updating more onerous than it needed to be. Related to this was the use of software and tools to manage specific tasks and produce content (leading to uncertainty over who controls access to the data), and unexpected changes to pricing structures.

***Information challenges.*** ‘Overwhelmed’, ‘exhausted’ and ‘fatigued’ were just some of the words used to describe members of guideline groups faced with selecting and appraising studies that were potentially relevant to their guideline. In one guideline (that included nearly 300 recommendations) the number of abstracts to screen for the most recent five-year update compared to the previous seven-year period increased by 250 per cent, amounting to over 100,000 abstracts.

*“Because it was such an enormous piece of work, there were hundreds and hundreds of questions, and I think what we tried to do was, frankly, just too big for human beings to do.” [P7]*
*“Well, I mean, all the citation management was done within Reference Manager at the time. There was a lot of Excel. I'm just trying to think back to what we did – it was all very analogue, lots of paper.” [P8]*

None of the respondents had used automation to facilitate citation screening or study appraisal but could see the potential for efficiency gains and were keen to learn more.

*“In a perfect world we’d get a machine to look at those abstracts… These days the technology does exist – we should be exploring it – and that would have saved months of human endeavour. It mightn’t be perfect but it’s probably as perfect as a human.” [P9]*

One respondent commented on the twin challenge of having an abundance of evidence for some questions and yet limited evidence for others, making the task of deriving recommendations equally challenging whichever the scenario.

***Publishing and dissemination arrangements.*** Publishing options for guidelines are constantly evolving and cover both traditional print and newer digital media. Despite innovations in publishing, several respondents perceived printed guidelines to be the preferred format of their users; this was especially so for guidelines that serve rural and remote practitioners, who are reluctant to switch to digital-only when reliable online access can’t be guaranteed. This potentially restricts the kinds of publishing and dissemination options available to guideline groups, even if the technical capacity and desire exists to do so.

*“We get a lot of people saying ‘I'm doing a remote placement and I've got your books but they're too heavy to carry’ – they’re around five kilos and if you're flying in a small plane, that's half your luggage quota gone. The challenge for us is we target remote areas – internet access isn't necessarily good and so a website isn't necessarily useful.” [P10]*

While two of the largest groups, responsible for comprehensive, all-of-condition guidelines, only publish online, this is the exception. Most developers provide an online version of their printed guidelines in PDF format – *“a thick PDF you’ve got to wade through”* – but that is often the extent of the digital functionality.

*“We had a big effort to make our website mobile friendly so it looks ok, but the ultimate thing is that we are still down to PDFs. It’s really very tricky.” [P5]*

The challenges some guideline groups face in going beyond traditional dissemination formats include constraints due to existing technology platforms (one group was still running Windows 7); variations in connectivity across jurisdictions (metropolitan versus rural); limited capacity to influence technology decisions (e.g. government decisions); and few resources to spend on publishing after the synthesis effort. Nevertheless, there is a broad willingness to embrace more responsive and digital publishing models.

*“We'll be really pushing the electronic. …We want to make it more electronic and more responsive.” [P8]*
*“We do want to move more online … to be more responsive.” [P11]*

It was also acknowledged that any new publishing formats are likely to be in addition to what is currently offered, further stretching constrained resources. For groups developing guidance that is more targeted at patients and carers, social media is another form of dissemination being considered.

*“We're also looking at more innovative ways of disseminating the content for consumers and carers as well... There's an incredibly huge role for social media and electronic media that we can use to engage consumers rather than the old fashioned printouts.” [P8]*

***Updating.*** Of all the challenges with the current process of guideline development, updating guidelines generated the most discussion. For some respondents, updating was a looming challenge that was either not yet an issue or had been deliberately put to one side until after the completion of the current edition of the guidelines.

*“But with these [guidelines], because it was short-term funding, we didn't really think that much ahead.” [P12]*

Many reflected on the realisation that updating should be a key consideration in the planning and conduct of any guideline. One respondent suggested that updating should be the first item on the agenda for any guideline group about to embark on the development of a new guideline, not least because of the implications for publishing arrangements.

*“Updating would have driven decisions about whether [the guidelines] were produced electronically or in print.” [P7]*
*“We were very diligent about thinking of uptake and implementation right at the outset … but what wasn’t front and centre of our minds was how the hell were we going to update these monsters once they were developed.” [P7]*

For the more-established guideline groups – those who have been through one or more cycles of guideline development and revision – how best to approach updating was an increasing focus of their guideline management. Conscious of the expense, time and personnel involved in producing updated editions of guidelines, respondents were exploring or implementing alternatives to conventional updates. However, as one respondent noted, willingness to move in this direction is not sufficient without the rest of the guideline apparatus being in place.

*“A restriction of working within [named organisation] is the technology is not there. We don’t have the funding for anything innovative, even to trial. Is it worth investing in regular updates if we can do nothing or we don’t have the capacity to incorporate them?” [P5]*

### Towards a new approach for developing guidelines

In considering what the future holds for guideline production, respondents focused discussion around improving efficiencies, accessing appropriate expertise and finding alternative approaches to updating. Several respondents recognised that adopting a more flexible model of updating guidance was not only desirable but also essential for ensuring sustainability of the whole guideline effort.

***Efficiencies in searching, screening and data extraction.*** Many respondents had attended meetings at which several new tools and technologies to support evidence synthesis had been presented and discussed, and were thus familiar with the potential of these technologies to accelerate guideline development processes, particularly around the time-intensive aspects, such as screening and data extraction. For scenarios where eligibility criteria around study design are clear (e.g. focus on systematic reviews and randomised trials), several respondents were aware of tools that classify citations according to the probability of being relevant or irrelevant, and of the potential time savings they offered.

*“If there were ways of text mining abstracts to be able to get a much quicker decision – ‘Is this abstract going to be relevant?’ – I think that would be wonderful.” [P13]*

None of the respondents had yet used these technologies, such as machine learning or crowd-sourcing, so while there was enthusiasm for experimenting to minimise screening workload, acceptability was an issue. Reservations were raised about what might be missed by relying on automation, as well as the loss of immersion in a topic that comes from iteratively screening the literature.

*“My concern … is that when you exclude a study, it just goes into an excluded studies bucket and you can’t really get it back out.” [P8]*
*“I think it's really important you get people doing the screening who really understand the context of the guideline. […] For intervention topics it can be a lot more straightforward but, like I say, for psychosocial screening questions and risk factor assessment type questions, it's much more nuanced.” [P8]*
*“If I was crowd-sourcing the screening I would be very concerned about the potential for excluding studies that should be included.” [P8]*

Like screening, the tasks of extracting data and assessing the validity of studies can be very resource-intensive. Several respondents were aware of systems and tools that could automate risk of bias assessments and aspects of data extraction but were yet to use them.

*“It would be very interesting to see if that [automation] could help with data extraction, etc. … which is time-consuming and quite taxing … reading hundreds of papers, it’s quite difficult at times. I think it would make a huge difference to our time and our resources.” [P14]*

***Synthesis and GRADE expertise.*** There were several steps in the evidence synthesis process where respondents felt they could benefit from help. Many respondents emphasised their lack of research synthesis expertise, whether among in-house staff or among guideline working group members. This issue varied depending on whether groups saw their primary role as translating evidence to produce practical guidance or doing the evidence synthesis. Those guideline groups that conduct their own evidence syntheses would like to access external specialists to conduct these in a timely and efficient way.

*“There will be occasions when we will need to do our reviews from scratch. I think that's where we would like to have support where we can.” [P11]*

Many commented on the rapid evolution of methods to assess the certainty of evidence and derive recommendations in guidelines, and were grappling with the transition from previous rating systems, such as the NHMRC’s FORM, to the more commonly used GRADE that NHMRC has now adopted as its preferred system when endorsing guidelines. The use of GRADE for deriving recommendations was cited as one area where guideline members do not have the time to undergo training and there is a lack of internal capacity to deliver it.

*“If we can access software or any kind of expert organisation that can help us to do systematic reviews, and as part of the GRADE process, that would be extremely helpful for us. That's currently a pretty intensive process and we don't have the resources to do it in house.” [P11]*
*“We'd like to educate our members as we move to GRADE. We'd like them to know what are the different terminologies that are used and get them to understand the differences in how you GRADE evidence and how you read the evidence. … So that when they start seeing these recommendations they fully understand why we say moderate evidence rather than the existing 1B or 2B.” [P12]*

For more experienced guideline developers, the transition to GRADE and using the software to produce the evidence tables was less of an obstacle. One respondent when asked if they felt adequately supported in how to use GRADE said:

*“I think so. I mean we just read the papers, looked at GRADEPro, watched some webinars and that was enough. I don't know what more benefit would have been to have had face-to-face training. They were quite good with email fortunately; if we had any questions and we emailed them, someone would always get back to us fairly quickly.” [P1]*

***Smarter use of existing evidence and closer Cochrane links.*** In the face of funding and time constraints, the necessity of pragmatic approaches to guideline development was frequently raised by participants. The abundance of guidelines produced globally gives groups more options when considering adapting existing high-quality evidence-based guidelines. Given the high costs of conducting a systematic review relative to the resources available for the guideline as a whole, guideline groups were understandably keen to identify cost-efficient, pragmatic approaches that achieved good outcomes without compromising quality or reliability.

*“We just don't have the resources to undertake systematic reviews … so we adopt a much more pragmatic approach to how we develop our guidelines.” [P12]*
*“There are times when we will develop recommendations from scratch, but that is quite rare and that's usually when we have grants and a lot of resources to support us.” [P11]*

The proliferation of published systematic reviews in recent years increases the likelihood that reviews already exist that fully or partially address questions in guidelines.

*“There's been an enormous volume of evidence published since the last guideline and so a lot of it has just been about having a really sensible approach to the update, and very much with a view to finding existing systematic reviews.” [P1]*

As groups look to adopt efficiencies in how their guidelines are developed and kept up to date, there was a clear role for Cochrane reviews to inform recommendations. However, while one respondent had contracted Cochrane to update a review, in general there was limited contact with Cochrane groups to help prioritise reviews for updating.

*“If there's an existing Cochrane review or an existing guideline that's used Cochrane methods – the NICE guidelines meet that criterion – then we use those as our foundation reviews. Just update those.” [P8]*

One guideline developer commented on their positive experience with using Cochrane reviews when they were reasonably up to date, but also on their frustration when they were several years old or only partially reported outcomes relevant to the guideline. This necessitated updating searches, going back to the primary studies and doing GRADE assessments, so that it was sometimes more efficient to ignore the existing Cochrane review and start again.

*“It would be great if you have found your Cochrane review and it's three years out of date and you'd like it updated, that would be fantastic. I guess the challenges are ones around timing and […] can it fall within the overall timeframe of the guideline. The other issue is around scope and […] what studies are included or excluded and what happens when the guideline group doesn't agree with the Cochrane review group.” [P8]*

One guideline group, that had drawn extensively on over 75 Cochrane reviews to inform their recommendations, felt that closer links with the respective Cochrane group (e.g. to align with priorities around updates or new reviews), as well as other guideline groups internationally, would be a much smarter way of collaborating.

Another guideline developer that makes use of existing systematic reviews was cautious about the reliability and variable quality of many so-called systematic reviews now being published.

*“Need to be sceptical about the methods others have used to do their systematic reviews. Some are not good quality.” [P15]*

***New approaches to updating.*** Several ideas were raised in relation to alternatives to the one-off guideline update. They included implementing an evidence notification system for hot topics, rather than relying on ad hoc approaches involving content experts, even if these specialists typically know if papers have been published that will impact on existing questions and recommendations. Similarly, an automated signalling service or horizon-scanning process would be a systematic way of identifying newly available research.

*“The idea of more automatically checking what's out there in the literature and bringing the results back would be very helpful, and may also to some extent resolve my concerns about the wrong sort of human influence [selectively picking their favourite studies].” [P4]*

Since the strength of the evidence underpinning recommendations varies, being able to ‘switch off’ questions because the evidence is highly stable and instead focus on those questions where there is uncertainty, was proffered as an efficient approach to adopt but, in contrast to the initial guideline development process, the guidance around updating was less clear.

*“I think we're really a little bit ad hoc, we don't have any kind of notification system.” [P14]*

The absence of a formal or consistent process for determining where to focus updating efforts was evident across the guideline groups. Some groups had already moved towards having dedicated updating staff (screening records, identifying new questions) and could see the benefits in using PICO-based tools (that use machine learning to determine the relevance of publications for particular topics) to streamline this process.

A cautionary note was struck by some respondents who expressed mixed feelings toward adopting a continual updating process. One concern was the impact on users of too-frequent changes to guidance, as well as how guideline groups would manage communication around these changes. Another was the risk of selectively identifying studies on a prospective basis instead of conducting comprehensive searches. Such a process would need to be transparent and require the dispassionate application of pre-specified criteria.

*“I have mixed feelings about the continuous update process, I think its strength is that the guidelines aren't getting out of date by the time the five-year, or whatever interval is chosen, comes up. On the other hand, there is a danger with the continuous update process that it gets triggered when somebody's favourite paper gets published… The process is meant to be more dispassionate, more arms length than that.” [P4]*

Several guideline developers expressed wariness in going back to their content experts with requests for ongoing involvement when those experts were already ‘extraordinarily fatigued’ by the push to produce the most recent update to the guideline. The resources and goodwill required to sustain advisory groups in a more responsive ‘living guidelines’ model have yet to be explored.

## Discussion

Clinical practice guidelines are an essential part of modern health care, helping to translate research into practice, inform policy decisions and improve patient outcomes. Their existence across all areas of health demonstrates their importance in supporting clinicians, patients and managers in making health-related choices. However, substantial resources and organisational commitment are required to produce and maintain guidelines. The Institute of Medicine contends that trustworthy guidelines should be informed by a systematic review of the evidence, follow explicit and transparent processes to minimise bias, and provide ratings of both the certainty and strength of recommendations ^1^. Guidelines should also be revised “when important new evidence warrants modifications of recommendations” ^1^. Even without the burden of keeping recommendations up to date, producing guidelines that meet accepted standards is a resource-intensive endeavour, requiring technical expertise, significant institutional support and the abundant commitment of guideline panel members.

In this study we explored the experiences of a diverse sample of Australian guideline developers and sought to understand their views regarding new ways of managing their guideline evidence needs. Through semi-structured interviews, we identified several challenges: limited technical expertise; constrained funding and timelines; interactions with external contractors; addressing information challenges; modernising publishing arrangements; and updating guidelines. Our findings reflect and expand upon the challenges facing clinical guidelines in Australia that were identified in a discussion paper from Australia’s NHMRC ^3^. Three of these link directly to what we found: lack of capacity (methodological expertise); lack of investment in information technology (widespread existence of manual systems, and paper or pdf publications); and obsolescence (keeping guideline recommendations up to date). This last challenge was seen as critical for several guideline developers we interviewed.

### Capacity

Systematic reviews that rigorously identify, appraise and synthesise relevant evidence to answer clinical questions are fundamental to evidence-based guidelines. Producing these systematic reviews requires guidelines developers to be, or to have access to and funding to support, “skilled groups of professionals with expertise in the methodology of evidence synthesis, experience in the application of these methods and the design, conduct and interpretation of systematic reviews” ^3^. Our findings concur with those of the NHMRC who identified that Australia lacks “the necessary critical mass of expertise and experience in the design and conduct of systematic reviews”, and that this is a crucial barrier to evidence-based guideline development. Our participants noted that a range of strategies are needed to overcome this challenge, including training to build workforce capability (e.g. GRADE Centres to provide support in key methods), establishment of consistent standards and approaches that are widely adopted, and which facilitate development of skills that are transferable across guideline projects (e.g. NHMRC guidelines for guidelines) ^11^ and exploring effective methods of international collaboration to enable reuse of evidence syntheses.

### Information technology

The sustainability and updating of evidence-based guidelines may be enabled by various technological developments that can maximise efficiency, especially for tasks like searching and screening that are most amenable to automation. Within Cochrane, for example, combining machine learning with crowd-sourcing has resulted in a high-performing study classifier for identifying randomised trials being implemented in review workflows and facilitating living systematic reviews ^12^. The guideline developers interviewed for this study were aware that these kinds of new technologies were becoming more routine and were keen to see how they could be used to save time and resources. However, as a recent survey among systematic reviewers has shown ^13^, uptake of these tools is impeded by several barriers, including mismatch to workflows, licensing, steep learning curve and lack of support.

### Keeping guidance up to date

Part of the rationale for developing systems that can handle more flexible approaches to updating is the phenomenal growth in global research output ^14^. Research synthesis, a discipline that evolved partly in response to the increase in primary research, is itself susceptible to the same trends. For example, around 25,000 systematic reviews are added to the Epistemonikos database annually and monthly registrations of systematic reviews in PROSPERO, the international prospective register of SRs, have increased 10-fold over the past five years ^15^. The challenge for guideline developers is clear: how can guidance feasibly be kept up to date when faced with this continual onslaught of evidence.

Not surprisingly, the three-to-five-yearly comprehensive guideline update is increasingly seen as impractical, and in some cases has already been abandoned in favour of more ‘living’ approaches, acknowledging that feasibility and sustainability are key issues. As a consequence of the various process constraints described in this paper, there is a strong desire for a guideline development model that explicitly recognises current limitations and invests in smarter, more efficient ways of maintaining and updating guidance.

### Towards living guidelines

Although the concept of living guidelines is not new, their uptake has been limited by practical considerations. A survey in 2009 of 40 guideline developers showed that while a majority of institutions supported the concept of living guidelines, they were regarded as very labour-intensive and requiring extra resources ^16^. In a 2014 review of guidance for updating provided in methodological handbooks for guideline developers, Vernooij found “the majority do not provide guidance for the literature search, evidence selection, assessment, synthesis, and external review of the updating process” ^17^. In the same year, in a systematic review of methods for updating clinical practice guidelines, partial updating was deemed more appropriate because it could be tailored to the different needs of the topic (i.e. dynamic versus stable evidence base) ^18^. The review concluded that guideline developers should “implement a systematic updating procedure that includes an ongoing monitoring system”. 

New, more dynamic approaches to updating guidelines would require the development of systems and tools to manage the flow and identification of potentially relevant new evidence, prioritise recommendations for surveillance, as well as establish new processes for managing the expert working groups involved in reviewing the evidence and revising the recommendations.

### Strengths and limitations

We applied purposive sampling to ensure we captured the perspectives and experiences of a variety of guideline developers. Groups represented in our interviews ranged from organisations responsible for substantial national programs of guideline activity through to smaller, ad hoc guideline groups convened to cover more narrowly-focused topics. The themes that emerged from the interviews, especially around issues affecting the production and updating of guidelines, were often common to all guideline groups regardless of size, longevity or funding. The in-depth and semi-structured nature of the interviews allowed for a richer understanding of the evidence needs facing guideline groups than could have been achieved through surveys or questionnaires. Although the inclusion of Australian-only guideline producers may limit the applicability of the findings, our informal interactions with guideline groups internationally often highlight similar themes, suggesting these findings are generalisable to other settings.

### Future research

Trustworthy clinical practice guidelines are an important knowledge translation tool but the challenge of producing and then keeping these guidelines up to date has been a perennial one for guideline developers. The concept of a ‘living’ guideline in which new evidence is incorporated as it is identified is one that is gaining momentum ^19^. This has partly been enabled by the growing popularity of online platforms to manage the conduct of evidence syntheses, as well as innovations in linked data and machine learning. For example, machine learning has several applications for guidelines, including the development of classifiers to automate the retrieval of relevant research and to model the risk of conclusions of systematic reviews changing ^20^.

Collaborations between guideline groups internationally may also increase the feasibility of maintaining living guidelines. In a partnership between the Stroke Foundation (Australia) and Cochrane Australia, we are aiming to support the translation of health research into practice by piloting living guidelines for stroke management ^7^. A similar project is underway in diabetes where four organisations have come together to pilot living recommendations in two priority areas of diabetes care ^21^. As well as evaluating the feasibility of processes such as continual evidence surveillance and rapid synthesis updates, both these initiatives address the challenges of obsolescence and lack of investment in information technology that were highlighted in the NHMRC report on the state of clinical guidelines in Australia ^3^. Future research should also evaluate the relative burden (costs, time, effort) of more continual approaches to updating compared to the stop-start nature of intermittent updating.

## Conclusions

Our research has defined the key challenges and needs faced by Australian guideline groups in developing rigorous evidence-based guidelines. Aside from the constant challenge of managing financial constraints, we identified several critical needs, including acquiring appropriate methodological expertise; investing in information technology; coping with the proliferation of research output; feasible publication and dissemination options; and keeping guidance up to date. For the guideline effort to be sustained, technological innovations (around platforms, tools, services and data) need to drive the efficiency of guideline development processes so that guideline developers can adopt more flexible, sustainable and still rigorous approaches to updating guidance.

## Data availability

### Underlying data

Guideline developers agreed to participate in the study on the basis of individual and organisational anonymity. The content of the audio recordings and transcripts contain information that would identify individuals and organisations. As a consequence, access to the audio-recordings and transcripts of the participant interviews, together with the content analyses, is unavailable.

### Extended data

Open Science Framework: Australian guideline developers: needs analysis. https://doi.org/10.17605/OSF.IO/H56TF ^10^.

This project contains the following extended data:

- McDonald_AusGuidelines_interview_guide.docx (interview guide used in this study).

### Reporting guidelines

Open Science Framework: COREQ checklist for “Towards a new model for producing evidence-based guidelines: a qualitative study of current approaches and opportunities for innovation among Australian guideline developers”. https://doi.org/10.17605/OSF.IO/H56TF ^10^.

Data are available under the terms of the Creative Commons Attribution 4.0 International license (CC-BY 4.0).
