# Supplementary material for: Towards a new model for producing evidence-based guidelines: a qualitative study of current approaches and opportunities for innovation among Australian guideline developers
Source: F1000Res. 2019 Jun 24;8:956. [Version 1] doi: 10.12688/f1000research.19661.1 (PMC6738187; doi:10.12688/f1000research.19661.1)
Supplement: Peer review history

# Peer review: Reviewer response for version 1

**DOI:** 10.5256/f1000research.21564.r53163

**Published:** 2019-09-10

**Authors:** Amanda Leach

**Affiliations:** 1 Menzies School of Health Research, Charles Darwin University, Darwin, NT, Australia

Please note that I am not a qualitative researcher so I have not made comment on these methodological matters.

 This report on the experience of 16 Australian Guideline developers in Australia is a very useful reality check for those embarking on, or responsible for updating, Clinical Practice Guidelines.

 I agree with reviewer Martin that the purposive sampling from “those who attended NHMRC-hosted forums” is likely to introduce bias. The authors could provide details of how many developers attended these forums, and potential bias due to familiarity of interviewees with interviewers. It would be of interest to interview developers who were not seeking NHMRC endorsement.

 The article covers the challenges of data extraction and synthesis well, exploring participant views on potential solutions including technologies such as machine learning. Whilst these are designed to improve efficiency, potential compromises to reliability of Guidelines should be highlighted as an area for future research.

 I would have liked to have learned more about efficient methods for disseminating guidelines or updates and monitoring the uptake and use of new Guidelines and updates, as well as for opportunities for users to score utility of the Guideline. There is potential for data to be collected on citations, number of digital downloads, the density of hits could identify hot and cold spots where and when Guidelines are being utilised and where and when they are not.  Leading to targeted efforts to improve uptake in ‘cold spots’.

 Updating was also very interesting and important – targeting searches towards clinical queries where there is low confidence and having questions that could be ‘switched off’ is a pragmatic option. One of the issues with updating is how to handle assessing the impact of updates on Guideline recommendations – keeping a review group over time who can determine whether an update changes current recommendations or changes the level of certainty or confidence in a recommendation, and what should be ‘published’. This is perhaps briefly touched on by “… without the rest of the guideline apparatus being in place.” When the evidence is low quality, new data could have a significant impact on a recommendation. In this instance there is also potential for recommendations to ‘flip-flop’ over time, leading to frustration and perhaps distrust of the Guideline.

 The article raises critical and urgent need for Australia to invest in research to answer the many challenges set out by the respondents.

 I don’t have specific recommendations for change, and look forward to reading more on progress in this space. Without such progress there will surely be a threat to quality of health care in Australia.

I confirm that I have read this submission and believe that I have an appropriate level of expertise to confirm that it is of an acceptable scientific standard.

# Peer review: Reviewer response for version 1

**DOI:** 10.5256/f1000research.21564.r50339

**Published:** 2019-08-27

**Authors:** Tracy Merlin

**Affiliations:** 1 Adelaide Health Technology Assessment (AHTA), School of Public Health, University of Adelaide, Adelaide, SA, Australia

This paper is a well written and thoughtful analysis of guideline developers' views on the challenges associated with guideline development in Australia. Many of the issues that were raised resonated with my experience of guideline development in Australia.

 I note two points that may have limited the applicability of the conclusions that have been drawn.

1. The study was jointly funded by Cochrane Australia and the NHMRC and the sampling strategy was purposive and drawn from attendees at NHMRC guideline development forums. It is therefor possible that the views that have been ascertained primarily relate to developers' experiences with developing NHMRC guidelines, as opposed to other guidelines that do not have NHMRC endorsement. I am aware of several guideline development groups that choose not to receive NHMRC endorsement in Australia because they find the guideline development requirements of the NHMRC overly restrictive. It is possible that these guideline developers would have had different perspectives on these challenges.
2. It would have been helpful to know the degree of experience in guideline development of the different study participants (i.e. the number of guidelines completed) and whether their views varied as a consequence. This was touched on when discussing the theme of "Synthesis and GRADE expertise". I would have liked to know whether it emerged with any other themes. This would help with understanding whether the themes identified are generalisable to all types of guideline developer. However, I do note that there was variation in size, longevity and funding of the different guideline groups interviewed.

The paper has concentrated on machine learning, crowd sourcing and automation as being possible solutions to the need to maintain high standards (good quality systematic reviews) but done in a way that would be time and cost-efficient. It was clear from the Results that although these methods are appealing in concept, very few of these methods have been trialled by guideline groups. It might be useful to reflect on this in the Discussion. What sort of time and cost efficiencies have been empirically found - to date (as I know the field is moving rapidly) - with these sorts of solutions?

 There was a short section on pragmatic review solutions in the Results section but this was not picked up in the Discussion. Was there any discussion of rapid review methodologies by the study participants, and how these are being used? Or use of overviews of systematic reviews? Would the authors like to comment on the reliability of a well done rapid review versus a comprehensive systematic review, or discuss the empirical evidence reporting on the merits of these? Rather than adopting new technologies and IT to solve the labour and cost-problem, is the solution being more pragmatic about the evidence-base i.e. what is the additional yield from doing a systematic review that has canvassed numerous databases, and conducts independent duplicate screening, extraction and/or critical appraisal, over other types of systematic review?

 Some of the points raised concerning external suppliers seemed to fit better with the updating theme.

 Participants 7 and 8 seemed to have more quotes used in the paper than others. Were their points supported by other participants in the study?

 The points raised regarding living reviews were interesting, particularly regarding the need to have predefined criteria for doing proactive horizon scanning or updated searching, rather than triggering an update due to publication of a known new paper. There is less room for selection bias if the process is standardised.

 It was encouraging to see this sort of research being done. The authors are to be congratulated on reflecting on these issues and considering how evidence based guideline methodology may be improved upon.

I confirm that I have read this submission and believe that I have an appropriate level of expertise to confirm that it is of an acceptable scientific standard.
